# Accuracy and concordance of measurement methods to assess non-adherence after renal transplantation - a prospective study

**DOI:** 10.1186/s12882-020-01781-1

**Published:** 2020-03-31

**Authors:** Marietta Lieb, Tobias Hepp, Mario Schiffer, Mirian Opgenoorth, Yesim Erim

**Affiliations:** 1 Department of Psychosomatic Medicine and Psychotherapy, Friedrich-Alexander-University Erlangen-Nürnberg (FAU), University Hospital of Erlangen, Schwabachanlage 6, 91054 Erlangen, Germany; 2 Institute of Medical Informatics, Biometry and Epidemiology, Friedrich-Alexander-University Erlangen-Nürnberg (FAU), Erlangen, Germany; 3 Department of Nephrology and Hypertension, University Hospital of Erlangen, Erlangen, Germany

**Keywords:** Adherence, Accuracy, Renal transplant recipients, Measurement methods, Electronic monitoring, Self-report, Physicians’ estimates, IS trough level variability, Prospective study

## Abstract

**Background:**

Non-adherence (NA) to immunosuppressants (IS) among renal transplant recipients (RTRs) is associated with higher risk of allograft rejection, graft loss, and mortality. A precise measurement of NA is indispensable, although its prevalence differs greatly depending on the respective measurement methods. The objective of this study was to assess the accuracy and concordance of different measurement methods of NA in patients after renal transplantation.

**Design and methods:**

This was a single-center prospective observational study. At baseline (T0), NA was measured via physicians’ estimates (PE), self-reports (SR), and tacrolimus trough level variability (CV%) in 78 RTRs. A Visual Analogue Scale (VAS, 0–100%) was applied both for SR and PE. In addition, we used BAASIS© for SR and a 5-point Likert scale for PE. NA was measured prospectively via electronic monitoring (EM, VAICA©) during a three month period. Meanwhile, all participants received phone calls in a two week interval (T1-T6) during which SRs were given.

**Results:**

Seventy-eight RTRs participated in our study. At t0, NA rates of 6.4%, 28.6%, and 15.4% were found for PE, SR, and CV%, respectively. No correlation was found between these methods. During the study, the percentages of self-reported and electronically monitored adherence remained high, with a minimum mean of 91.2% for the strictest adherence measure (Timing Adherence ±30 min). Our results revealed a moderate to high association between SR and EM. In contrast to PE and CV%, SR significantly predicted electronically monitored adherence. Overall, a decreasing effect of electronically monitored adherence was found for both taking and timing adherence (±2 h, ±30 min) over the course of the study.

**Discussion:**

The moderate to high concordance of SR and EM suggests that both methods measure NA equally accurately. SR seems to be a method that can adequately depict electronically monitored NA and may represent a good and economical instrument to assess NA in clinical practice. The increased adherence at the beginning of the study and its subsequent decrease suggests an intervention effect. Surveillance of IS intake via EM with intermittent phone calls could improve adherence on a short-term basis. To establish long-term effects, further research is necessary.

## Background

Adherence is defined as “the process by which patients take their medications as prescribed” [1]. In case of Non-Adherence (NA) to the immunosuppressive (IS) regimen after renal transplantation, detrimental effects on graft survival and functioning can occur. NA has been identified as a major contributing factor for acute cellular and chronic allograft rejection, graft loss, and mortality [2–5]. Nonetheless, a substantial number of renal transplant recipients (RTRs) are non-adherent to their IS medication, on average 35.6% of patients per year [6]. This prevalence differs greatly depending on the respective measurement method, leading to NA rates ranging from 1.6% to 58.7% [7]. A wide array of different NA measures has emerged in the past years including direct and indirect measures with various advantages and disadvantages. Direct measures comprise direct observation of medication intake, biomarkers (tracers consumed with drug), and measurement of IS trough level variability in the blood [8–10]. Indirect measures include pill counts, self-reports (diaries, questionnaires, interviews), collateral reports from family members or clinicians, prescription refills, and electronic monitoring [8, 9]. For measures of NA in RTRs, self-reports, collateral reports, and IS trough level variability are most frequently applied, due to their inexpensiveness and practicability [8].

However, collateral reports such as physicians’ estimates are mostly considered unreliable and biased [11, 12]. Although self-reports yield the risk of underestimating NA due to social desirability or memory bias [8, 13], they are viewed as inexpensive and practical tools for clinical settings [14–16]. In particular the self-report questionnaire BAASIS©, which includes the assessment of taking, timing, and dosing adherence, is a widely used and recommended instrument [15]. The IS trough level variability has also become a standard measure of patients’ adherence in clinical practice, since it reflects the patient’s consumption of immunosuppressive drugs [8, 17]. Especially the coefficient of variation is increasingly used and has been associated with rejection and poor renal graft outcome [18–20]. However, results can be influenced by several biological parameters (metabolic rates, drug interactions, etc.) and distort interpretation [9, 11].

Electronic monitoring (EM) has recently gained great popularity. It is often declared as the best estimate of NA [13, 15, 21], since it provides insight into the dynamics of both taking and timing adherence over a period of time [8, 15]. Although the utilization of EM as a method for monitoring immunosuppressive NA is on the rise and used in a variety of studies [22–27], the accuracy of this method is still being debated. Electronic malfunction, incorrect use, negative change in routine, but also an induced intervention effect resulting in improved adherence, are considered potential causes for imprecise EM measures [21, 28–31]. Studies on the correlations of EM with other adherence measures show mixed results. In a review on adherence in a general population, a high to moderate concordance between EM and self-reports could be found [32], whereas another review found self-reports to be systematically underestimating NA compared to EM [33]. Studies examining the correlations of different adherence measures in RTRs are sparse. One study found self-reported NA to be a significant predictor of electronically monitored NA during a three months course. However, it only showed a 26% sensitivity compared to EM [14]. Similar results were found in a study by Butler et al. [12]. Another study of Schäfer-Keller et al. [34] monitored immunosuppressive NA over a three months period. IS trough levels, self-reported NA, and clinician’s collateral reports showed significant but low correlations with EM. The concordance of other adherence measures depicts equally heterogeneous results. Liu et al. [16] found self-reported NA to be associated with serum concentration fluctuation for tacrolimus but not cyclosporine. Similar results were found by Lalic et al. [35], while other studies found no correlation at all between self-reports and IS trough levels [10, 34]. Equally, no associations were detected between collateral reports and self-reports [11, 34, 36], nor between physicians’ estimates and the variability of trough levels [11, 34]. Some research also recommends combining several measurement methods to increase sensitivity [9, 15, 34, 37]. Overall, there still seems to be some lack of consensus on how exactly measures of NA correlate with each other and which approach is the best. Especially in RTRs, evidence seems scarce. With this study we aim to close this gap by prospectively examining the concordance of different adherence measures in RTRs. To our knowledge, no study has yet examined both self-reported and electronically monitored immunosuppressive NA in a longitudinal design.

## Methods

### Objectives of the study

The primary objective of this work was to investigate the correlation between different measurement methods of immunosuppressive NA during the phase of medication implementation [1, 38]. Besides IS trough levels and physicians’ estimates on NA, special attention was paid to electronically monitored and self-reported NA. The following issues were addressed: 1) To what degree do the relevant measurement methods interrelate at the beginning of the study? 2) Does electronically monitored NA coincide with patients’ self-reports during the course of the study? 3) Can self-reported NA, physicians’ estimates and/or trough levels predict the degree of electronically monitored NA during the study? 4) How does the measured NA change over time? Does the study design induce an intervention effect on NA?

### Study population and recruitment methods

This work is part of the APT (Adherence and Psychological Health after Transplantation) research project of the Department of Psychosomatic Medicine and Psychotherapy and took place in cooperation with the Department of Nephrology and Hypertension and the Department of Cardiac Surgery in Erlangen. Our sample was consecutively derived from RTRs attending their routine follow-up examination at the renal transplant outpatient clinic from March 2018 to April 2019. Potential participants were contacted via telephone prior to their regular appointment. If interested, study information and questionnaires were sent to the respective patients. RTRs aged 18 years or more who were at least six months post-transplantation and received tacrolimus (Advagraf© or Prograf©) as their prescribed IS medication were eligible. We specifically focused on medication implementation and excluded cases of initiation [1, 38]. Exclusion criteria encompassed insufficient German language skills, severe mental disorders, and/or cognitive impairments. Institutional ethic board approval was obtained from the Clinical Ethics Committee of the University Hospital Erlangen (Friedrich-Alexander-University, Erlangen-Nürnberg, FAU). Written informed consent was given by all participants.

### Data collection and measurement of non-adherence

#### Electronic monitoring

At enrollment, all participants received an electronic multicompartment pillbox (VAICA SimpleMed©) which allows the storage and organization of medication up to seven days and up to four doses a day. The patients’ medication plan for their main immunosuppressant (Advagraf© or Prograf©) was entered on the web portal of the corresponding device. We defined two time intervals of ±30 min and ± 2 h of the prescribed IS intake time. Pill extraction times were registered electronically and transferred to the respective patients’ web-based pillbox record. Reminder functions (visual and acoustic sounds) were disabled for all patients during the whole course of the study. To improve validity, participants were asked to keep log of incidents when the opening of the compartments did not correspond with medication intake (e.g. IS were extracted early and taken later) or when the medication was taken from another source (take-away box, pocket, purse etc.) [39]. To determine whether electronic monitoring might influence the patients’ normal adherence behavior [28, 29], we asked the participants to rate its subjective effect on a scale from − 10 (negatively influenced) to + 10 (positively influenced).

#### Self-report

For self-reports on IS intake, we used the Basel Assessment of Adherence to Immunosuppressive Medication Scale (BAASIS© [15]). It consists of four items on immunosuppressive NA (dose taking, drug holidays, timing adherence ±2 h, dose reduction) to be rated on a 6-point scale (0 = no/never, 5 = every day) referring to the last four weeks. Consent to at least one of the items is considered non-adherent (= dichotomous). Further, a total score of the four items results in a continuous adherence rating with scores ranging from 0 to 20 [40]. We used an altered version of this questionnaire as we added a further question on timing adherence, asking for a deviation of intake time of ±30 min. We further reduced the recall period to two weeks during the course of the study. In addition to the 6-point rating scale, we asked for the absolute frequency of deviations from the prescribed medical regimen. At the beginning of the study, we also asked each participant to rate his overall adherence on a 10 cm (0–100%) Visual Analogue Scale (VAS) (BAASIS© Interview).

#### Physician’s estimates

After the patients’ routine follow-up examination, the treating nephrologist was asked to assess the patients’ overall adherence on a 5-point scale (1 = very good, 5 = very bad). Patients who were rated less than good (> 2) where considered non-adherent. Similar measurement methods have been used by other researchers [11]. In addition, the patients’ general adherence was assessed on a 10 cm (0–100%) Visual Analogue Scale (VAS) to reach comparability with the patients’ self-reports.

#### IS trough levels

IS trough levels (Tacrolimus) are routinely measured during the patients’ follow-up examination at the outpatient clinic of the nephrologic department of the University Hospital in Erlangen. We used the IS trough level which was measured at the day of study enrollment as well as up to three available antecedent measures [10], irrespective of the individual interval each patient attended their follow-up visits. IS levels from several time points are considered more reliable than only one [11, 17]. We excluded successive measures that were taken during hospitalization. We then assessed the IS trough level variability (CV% [17]), which was previously found predictive of graft rejection, organ loss, and NA [18–20, 41]. If only one IS trough level was available and variability was not calculable, we excluded this case for this specific analysis. For the calculation of the CV% we first standardized IS levels by dividing each IS level by their respective target level, which is determined individually for each patient and can be changed by the physicians depending on its clinical course. For each standardized IS trough level, we calculated means (M) and standard deviations (SD). The CV% was then calculated by dividing SD through M and multiplied by 100. Higher CV%s reflect a higher degree of irregularity in IS trough levels [10, 17]. A CV% threshold of approximately > 30 was previously associated with poor renal allograft outcomes [20, 41–43] and non-adherence [19], therefore we categorized patients with CV% > 30 as non-adherent and CV% < 30 as adherent, respectively.

### Study design and measurement points

**Fig. 1:**
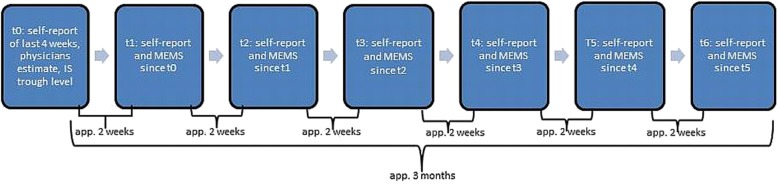
Timeline of study procedure

This is a prospective single-center observational study. Electronic monitoring took place for three months. During this time, each participant was called every second week and asked to give a self-report on potential NA of the last two weeks (Fig. 1).

At the end of the study, patients were asked to return the electronic pillbox and received a detailed feedback on their electronically monitored adherence behavior.

### Data management

Deficient data from the electronic pillbox were corrected via notes provided by the patients’ diaries. At the end of the three months EM period, the participants were asked to assess the consistency of diary and pillbox use on a 0–10 scale. If patients stated inconsistent use of the dairies and/or the pillboxes (ratings from 0 to 5) or if technical failure occurred over the whole study or a certain period of the study, the respective measurement points were eliminated from analysis [28, 29]. In cases of which patients were not using the pillbox for a certain period of time (e.g. travel, hospitalization, weekend etc.), we extended the individual study period for the respective time, if possible. If patients could not be reached via telephone in the two week interval, we extended this period to a maximum of three weeks. After four weeks, the respective measurement points were specified as missing.

EM-Imputations were used to replace single missing values. Missing values for EM, whole psychometric scales, and measurement points (due to drop-outs, eliminated measures etc.) were not replaced. The measurement points or number of patients included in the respective analyses are indicated. It has to be noted that Timing Adherence ±30 min (TM ± 30 min) includes all cases of Timing Adherence ±2 h (TM ± 2 h) while TM ± 2 h includes all cases of Taking Adherence (TA).

### Statistical analysis

For descriptive statistics, we depicted frequencies, mean values, standard deviations, and ranges. For group comparisons (Non-Responder Analysis), independent T-tests, Mann-Whitney-U-tests, and Chi^2^-tests were used. Analyses on the associations of measurement methods comprised Cohen’s kappa and the Intraclass correlation coefficient (ICC). Three linear regression models were conducted with the percentage of electronically monitored adherence (TA, TM ± 2 h, TM ± 30 min) as the outcome variable and sex, age, physicians’ estimates (VAS), self-reports (VAS), and IS trough level variability (CV%) as independent variables. To assess changes in probability of adherence over time, we dichotomized the electronically monitored adherence scores (for TA, TM ± 2 h, and TM ± 30 min respectively; 0: 100% adherent, 1: ≤ 100% adherent). Two alternative linear mixed models (linear and piecewise linear) were conducted for each variable. Data were processed and analyzed using the software SPSS 21 for Microsoft Windows©. Linear mixed models were carried out using the nlme-package [44] and the lme4-package [45] for R version 3.5.1. All results were interpreted on a significance level of *p* < .05. We used Bootstrap Confidence intervals with 10,000 iterations in case of severe deviations from the normal distribution.

## Results

Of 231 eligible RTRs, a total of 184 patients could be contacted by phone. 89 initially agreed to participate in our study. 11 dropped out before activating the pillbox or during the first week, one patient resigned during the first month, and another during the second month of pillbox usage (=NR). A total of 76 patients completed the study (=P). Reasons for not participating or drop-outs included sickness, lack of time, or impracticability of the pillbox. A Non-Responder Analysis (including drop-outs and refusals, *n* = 108) revealed no significant difference in age (P: mean = 55.64 years; NR: mean = 52.12 years; t = − 1.85, *p* = .065), year of transplantation (P: median = 2013; NR: median = 2015; U = 3452.00, p = .065) and sex (χ^2^ = 3.12, *p* = .077) between those who participated and those who did not. Sociodemographic and biomedical data of participating patients are displayed in Table 1.

**Table 1:** Sociodemographic and biomedical data

		All participants (*N* = 78)^a^
Age (in years; M, SD, range)		55.28, 11.52, 30–78
Sex (n, %)	Males	56 (71.8)
	Females	22 (28.2)
Graduation (n, %)	Intermediate school or less (< 12 years)	61 (78.2)
	High School or higher (> 12 years)	16 (20.5)
	No information	1 (1.3)
Marital Status	Single	11 (14.3)
	Married/in a relationship	60 (76.9)
	Widowed	5 (6.5)
	Separated/Divorced	2 (2.6)
Children	Yes	54 (69.2)
	No	22 (28.2)
	No information	2 (2.6)
Employment status	Employed (full or part-time)	30 (38.5)
	Unemployed/retired	41 (52.6)
	Other	3 (3.8)
	No information	4 (5.1)
Migration background^b^	Yes	5 (6.4)
	No (German)	72 (92.3)
	No information	1 (1.3)
Immunosuppressive Medication (n, %)	Advagraf© (once daily)	48 (61.5)
	Prograf© (twice daily)	30 (38.5)
Number of transplantations (n, %)	1	70 (89.7)
	2	6 (7.7)
	3	2 (2.6)
Type of renal graft (n, %)	Living	31 (39.7)
	Postmortem	47 (60.3)
Types of organs transplanted (n, %)	Single Kidney transplantation	70 (89.7)
	Dual Kidney transplantation	1 (1.3)
	Pancreas-Kidney transplantation	7 (9.0)
Last transplantation (in years; Median, SD, range)		2013, 4.21, 2001–2018

Note: a: includes the two patients who dropped out after the first and third month b: Migration background is defined as either immigrated personally or having at least one parent who has immigrated

### Prevalence of non-adherence at the beginning of the study (t0)

Physicians’ estimates ranged from 1 (very good) to 3 (moderate), with a total mean of 1.36 (SD: .60). 73 of the 77 patients (93.6%) were rated with good or very good (= adherent), whereas 5 patients (6.4%) were rated to have moderate adherence (= not adherent). On the Physicians’ Visual Analogue Scale (VAS), adherence ratings ranged from 73% to 100%, with a mean of 92.27% (SD: 5.47). 80.8% of all patients were rated to have an overall adherence of 90% or better. Self-reports (dichotomous assessment of BAASIS©) revealed NA in 22 patients (28.6%). 67 patients (85.9%) claimed not to have missed any dose during the past four weeks, whereas 10 (12.9%) said it happened at least once. No one claimed to have skipped several doses in a row. 59 patients (75.6%) stated that they always took their IS within a 2-h interval, whereas 18 patients (23.1%) said they deviated from it at least once. No one stated an autonomous reduction of dosage. 59 of our participants received the additional question on the 30 min intake interval. Of those, 18 (30.5%) patients never deviated from their intake time more than 30 min, whereas 22 (37.3%) delayed their intake once per months and 19 (32.2%) said it happened at least every other week or more often. On the patients’ VAS, self-reported adherence ranged from 10% to 100%, with a mean of 92.03% (SD: 13.51). 81.8% of all patients rated themselves to have an adherence of 90% or better.

The IS trough level variability (CV%) ranged from 4.4% to 64.5% with a mean of 21.1% (SD: 10.2). Of the 78 participants, 12 (15.4%) exhibited a IS trough level variability (CV%) > 30, 65 (84.3%) stayed below this threshold, while one patient (1.3%) was excluded from the analysis due to missing data. In conformity with the recommendation of *triangulation* in order to increase the sensitivity of measurement methods [9, 15, 34, 37], we combined the dichotomous scores of physicians’ estimates, self-report, and IS trough levels to attain a composite NA score. We found 31 (40.8%) patients who had at least one rating of NA in any of these three categories.

### Association of Measurement Methods at the beginning of the study (t0)

There was no association between the physicians’ estimates (VAS) and self-reports (VAS) of adherence (ICC = .196, *p* = .174). Similar results appeared regarding the dichotomous classification of adherence, revealing no agreement of the respective assessments: Only 5 (6.6%) patients were rated non-adherent by their treating nephrologist, while a total of 22 patients (28.21%) rated themselves as non-adherent. Of those, only 3 (13.64%) were classified accordingly by their physician, whereas the remaining 2 were rated adherent (k = .130, *p* = .108). Equally, IS trough level variability (dichotomous) was not significantly associated with physician’s estimates (k = .158, *p* = .120): Only 2 of the 5 patients rated as non-adherent had an increased IS trough level variability. Neither was the continuous CV% associated with the physicians’ estimates (VAS, ICC = −.003, *p* = .701). The dichotomous IS trough level variability was also not associated with the dichotomous self-rating of BAASIS© (k = .039, *p* = .715): Only 4 of the 22 who rated themselves non-adherent showed a CV% above the critical threshold. The continuous scores of CV% and self-report (VAS) did also not show any correlation (ICC = .014, *p* = .134). An overview of the associations between the different measurement methods at the beginning of the study (t0) can be viewed in Table 2.

**Table 2:** Association of adherence measurement methods at t0

	Self-report (VAS)	Physicians’ estimates (dichotomous)	Physicians’ estimates (VAS)	IS trough level variability CV% (dichotomous)
**Self-report: BAASIS©** **(4 Item Original)** **Dichotomous**	xx	k = .130, *p* = .108	xx	k = .039, *p* = .715
**Self-report (VAS)**	xx	xx	ICC = .196, p = .174	xx
**IS trough level variability CV% (dichotomous)**	xx	k = .158, p = .120	xx	xx
**Is trough level variability (CV%)**	ICC = .014, p = .134	xx	ICC = −.003, *p* = .701	xx

Note: *0 = adherent, 1 = non-adherent

### Prevalence of electronically monitored and self-reported non-adherence during the study (t1-t6)

During the course of the study (t1-t6), the NA rate measured by BAASIS© (patients who agreed to any of the four items, two weeks recall) varied between 9.3% (t2) and 20.5% (t6). 70.3% of all participants reported full adherence throughout the whole study. 29.7% claimed NA at least once during study course. Taking NA alone was stated by 1.3% (t3) to 5.6% (t5) of all patients. A deviation from the ±30 min interval, which included all incidences of taking and timing NA ±2 h, was confirmed by 38.2% (t1) – 51.4% (t5) participants and only 23.2% reported full timing adherence ±30 min during the whole study. When extending the recall period to 4 weeks, equal to the original BAASIS© (by adding t1 + t2, t3 + t4, and t5 + t6, respectively), the prevalence rates were as follows: 10.8% (t1 + t2), 17.1% (t3 + t4), and 26.8% (t5 + t6).

The percentages of self-reported adherence remained relatively high throughout the whole study. Self-reported TA showed a relatively stable mean with a minimum of 99.47% (±2.3) at t5 and a maximum of 99.8% (±1.2) at t2 with minimal ranges (85.7–100%) during the study (t1-t6). Drug holiday never occurred. The percentage of self-reported TM ± 2 h (including TA) ranged from 21.4% to 100% from t1-t6 while the mean percentage per measure stayed continuously high with a minimum mean of 97.7% (±9.4) at t6 and a maximum mean of 99.4% (±1.7) at t4. The percentage of self-reported TM ± 30 min (including TA and TM ± 2 h) displayed a wide range from 0% to 100% during study course, whilst the mean still only reached a minimum of 94.3% (±12.7) at t5 and a maximum 96.1% (±6.6) at t1. Dose reduction did not occur from t1-t5, but occurred in two cases (2.7%) at t6, whereas 97.3% complied with their prescribed dosage.

Electronically monitored adherence showed similarly high percentages. TA showed a minimum percentage of 85.7% (t6) with constantly high means ranging from 98.9% (±2.4) at t6 to 99.6% (±1.4) at t1. The percentage minimum of electronically monitored TM ± 2 h (including TA) was 61.9% (t2), whilst it displayed constantly high means ranging between 97.2% (±4.9) at t6 and 99.0% (±4.6) at t2. TM ± 30 min dropped to a minimum percentage of 0% (t4, t5). Means again remained high with a range between 91.2% (±14.9) at t6 and 94.0% (±19.1) at t2. Corresponding figures can be found in the Additional files (1, 2, 3, 4, 5 and 6).

### Associations of self-reported and electronically monitored non-adherence during the study (t1-t6)

The associations between self-reported NA and electronically monitored NA are based on absolute frequencies of the respective deviations and are depicted in Table 3. The correlations between Taking NA ranged from a fair correlation at T3 (ICC = .493) to a good agreement at T2 (ICC = .739). The correlations of Timing NA ±2 h ranged from ICC = .50 (T3, T6) to ICC = .895, while those for Timing NA ±30 min correlated poorly at T3 (ICC = .394) and very high at T4 (ICC = .905). In total, the correlation of methods was highest for Timing NA ±30 min (ICC = .879) and lowest for Timing NA ±2 h (ICC = .634). It has to be noted that the absolute frequencies (Total ∑T1-T6) of electronically monitored NA had constantly higher means than self-reported NA (Taking NA: .83 vs. .26, Timing NA ±2 h: 2.19 vs. 0.94, Timing NA ± 30 min: 8.44 vs. 5.8).

**Table 3:** Correlation of self-reported and electronically monitored non-adherence

		n	ICC			n	ICC
T1	Taking NA	76	ICC = .648, *p* < .001	**T4**	Taking NA	70	ICC = .642, *p* < .001
	Timing NA ±2 h	76	ICC = .625, *p* < .001		Timing NA ±2 h	69	ICC = .587, *p* < .001
	Timing NA ±30 min	67	ICC = .783, *p* < .001		Timing NA ±0 min	70	ICC = .905, *p* < .001
T2	Taking NA	69	ICC = .739, *p* < .001	**T5**	Taking NA	64	ICC = .603, *p* < .001
	Timing NA ±2 h	69	ICC = .895, *p* < .001		Timing NA ±2 h	64	ICC = .519, *p* = .001
	Timing NA ±30 min	63	ICC = .714, *p* < .001		Timing NA ±30 min	64	ICC = .808, *p* < .001
T3	Taking NA	71	Not computable*	**T6**	Taking NA	68	ICC = .493, *p* = .001
	Timing NA ±2 h	71	ICC = .500, *p* = .001		Timing NA ±2 h	68	ICC = .500, *p* = .003
	Timing NA ±30 min	78	ICC = .394, *p* = .018		Timing NA ±30 min	68	ICC = .836, *p* < .001
				**Total**	Taking NA	57	ICC = .741, *p* < .001
				**∑T1-T6**	Timing NA ±2 h	56	ICC = .634, *p* < .001
					Timing NA ±30 min	48	ICC = .879, *p* < .001

Note: We correlated the absolute frequencies of self-reported and electronically monitored non-adherence; Timing NA (±2 h) includes all cases of Taking NA, Timing NA (±30 min) includes all cases of Taking and Timing NA (±2 h). *no variation in data. NA = Non-Adherence

As depicted in Table 4, linear regression analyses revealed only self-reported adherence (VAS) as a significant predictor for the percentage of electronically monitored TA, TM ± 2 h, and TM ± 30 min, respectively.

**Table 4:** Linear regression analysis

Dependent Variable	Parameter	B	95% CI	*p*	R^2^
TA (Total)	Constant	94.172	87.642, 102.299	.000	.150
	Age	−.011	−.032, .014	.396	
	Sex	.129	−.272, .525	.546	
	CV%	−.006	−.021, .014	.432	
	Physician‘s estimates (VAS)	.038	−.041, .103	.415	
	Self-report (VAS)	.027	−.001, .041	.011*	
TM ± 2 h (Total)	Constant	88.732	74.577, 107.131	.000	.143
	Age	−.019	−.063, .027	.404	
	Sex	−.171	−1.026, .680	.701	
	CV%	−.022	−.060, .011	.178	
	Physician‘s estimates (VAS)	.052	−.119, .182	.567	
	Self-report (VAS)	.071	.017, .125	.006*	
TM ± 30 min (Total)	Constant	49.713	−4.291, 120.920	.183	.199
	Age	−.085	−.244, .066	.297	
	Sex	−.888	−3.854, 1.865	.556	
	CV%	.033	−.106, .162	.580	
	Physician‘s estimates (VAS)	.215	−.469, .783	.607	
	Self-report (VAS)	.313	.124, .443	.001*	

Note: estimates are based on 10,000 Bootstrap-samples, * *p* < .05, TA = Taking Adherence, TM + -2 h = Timing Adherence + − 2 h,

TM + -30 min = Timing Adherence, CV% = Coefficient of variability, PE = Physicians’ estimates, SR = self-report, VAS = Visual Analogue Scale.

### Effect of measurement and change of non-adherence over time (t1-t6)

**Fig. 2:**
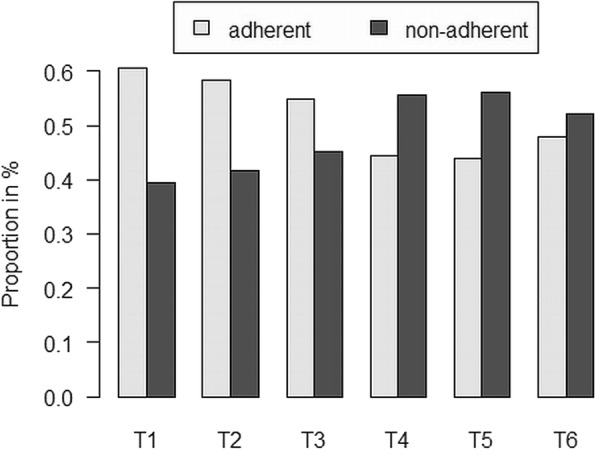
Percentage Frequency of Timing Adherence ±30 min – Electronic Monitoring (raw scores)

**Fig. 3:**
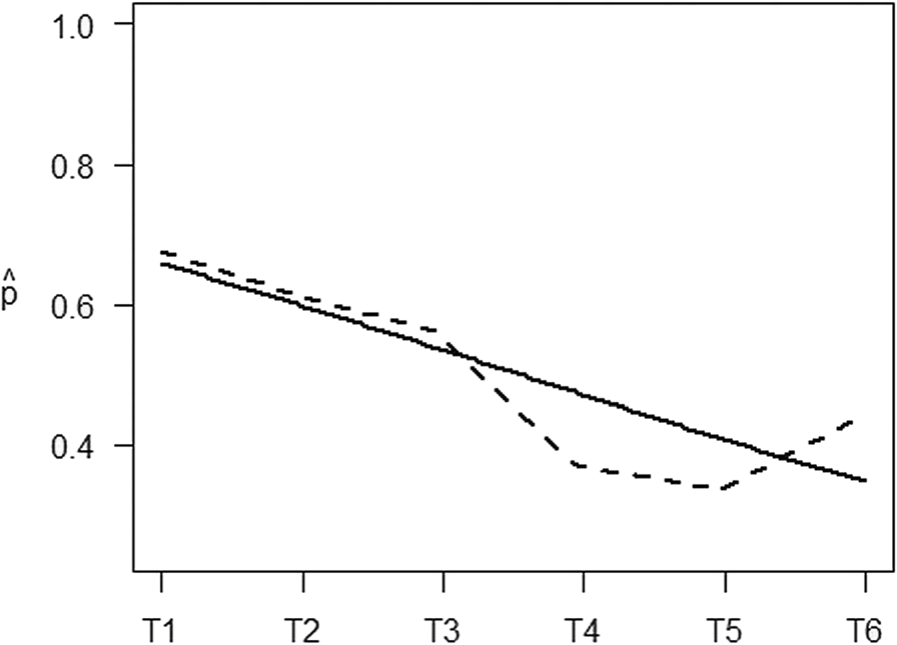
Estimated probability of Timing Adherence ±30 min – Electronic Monitoring

The linear mixed models confirmed a significant decrease in the probability of electronically monitored TA (*p* < .001), TM ± 2 h (p < .001) as well as TM ± 30 min (*p* < .001) during the course of the study (Table 5). Figs. 2 and 3 depict the steady decrease of electronically monitored TM ± 30 min. Although the piecewise linear model did not reach significance for any specific measurement points, there was a declining trend in the probability of electronically monitored TM ± 30 min from T3 to T4 (*p* = .07). A slight increase, however, could be witnessed from T5 to T6 (Fig. 2), indicating again a slight rise in adherence at the end of the study compared to T5. No significance could be reached, though (*p* = .31).

**Table 5:** Linear and piecewise linear mixed models on the change of adherence over time

Variable	Model	Parameter	Estimate (CI)	SE	z-value	*p*-value
TA – Electronic monitoring	LMM – with linear trend	Intercept Time	5.05 (3.32, 6.77)	0.88	5.74	<.001**
			−0.41 (− 0.65, − 0.172)	0.12	−3.38	<.001**
	LMM – with piecewise linear trend	Intercept	4.16 (2.49, 5.82)	0.85	4.90	<.001**
		Time ≥ T2	0.77 (−0.86, 2.40)	0.83	0.93	0.35
		Time ≥ T3	−1.13 (−2.72, 0.47)	0.81	−1.38	0.17
		Time ≥ T4	−0.01 (−1.31, 1.30)	0.67	− 0.01	0.99
		Time ≥ T5	−0.81 (− 2.07, 0.45)	0.64	1.26	0.21
		Time ≥ T6	−0.49 (− 1.61, 0.62)	0.57	− 0.87	0.39
TM ±2 h – Electronic monitoring	LMM – with linear trend	Intercept	3.21 (2.26, 4.15)	0.48	6.64	<.001**
		Time	−0.37 (− 0.55, − 0.20)	0.09	−4.13	<.001**
	LMM – with piecewise linear trend	Intercept	2.44 (1.51, 3.37)	0.47	5.17	<.001**
		Time ≥ T2	0.58 (−0.53, 1.70)	0.57	1.03	0.31
		Time ≥ T3	−0.96 (−2.06, 0.14)	0.56	−1.71	0.09
		Time ≥ T4	−0.25 (− 1.20, 0.71)	0.48	−0.50	0.61
		Time ≥ T5	−0.47 (−1.41, 0.47)	0.48	−0.97	0.33
		Time ≥ T6	−0.48 (−1.39, 0.43)	0.46	− 1.02	0.31
TM ±30 min – Electronic monitoring	LMM – with linear trend	Intercept	0.91 (0.15, 1.68)	0.39	2.35	<.05*
		Time	−0.26 (− 0.41, − 0.11)	0.08	−3.38	<.001**
	LMM – with piecewise linear trend	Intercept	0.76 (−0.04, 1.56)	0.41	0.41	0.06
		Time ≥ T2	−0.29 (−1.15,0.56)	0.44	0.44	0.50
		Time ≥ T3	−0.22 (−1.07, 0.63)	0.43	0.43	0.61
		Time ≥ T4	−0.79 (−1.64, 0.06)	0.43	0.43	0.07
		Time ≥ T5	−0.13 (− 0.99, 0.74)	0.44	0.44	0.77
		Time ≥ T6	0.45 (− 0.43, 1.33)	0.45	1.01	0.31
**Variable**	**Model**	**Parameter**	**Estimate (CI)**	**SE**	**t-value**	**Sign.**
Intervention effect – self-assessment	LMM – with linear trend	Intercept	1.81 (1.01, 2.62)	0.41	4.43	<.05*
		Time	0.02 (−0.10, 0.13)	0.06	0.29	ns
	LMM – with piecewise linear trend	Intercept	1.95 (1.13, 2.77)	0.42	4.66	<.05*
		Time ≥ T2	−0.14 (−0.82, 0.55)	0.35	−0.39	ns
		Time ≥ T3	−0.05 (− 0.74, 0.65)	0.35	− 0.13	ns
		Time ≥ T4	−0.15 (− 0.55, 0.85)	0.36	0.41	ns
		Time ≥ T5	−0.20 (− 0.90, 0.50)	0.36	− 0.56	ns
		Time ≥ T6	0.40 (−0.30, 1.10)	0.36	1.12	ns

Note: ** < .001, * *p* < .05, TA = Taking Adherence, TM + -2 h = Timing Adherence + − 2 h, TM + -30 min = Timing Adherence, LMM = Linear Mixed Model, ns = not significant

For the self-assessed intervention effect, no significant change over time could be confirmed. Its mean ranged between 1.7 (±3.6) at t2 and 2.3 (±3.8) at t6. All corresponding figures are available in the Additional files (7, 8 and 9).

## Discussion

### Prevalence of non-adherence and Association of Measurement Methods at the beginning of the study (t0)

This study prospectively examined the concordance and accuracy of different measurement methods of immunosuppressive NA in RTRs with special focus on self-reported and electronically monitored NA. In our study, the prevalence of self-reported NA was 28.6% when measured with BAASIS© at t0. Similar prevalence rates of self-reported NA can be found in other studies using BAASIS©, displaying ranges between 27% and 36.6% [10, 11, 46]. For the self-reported NA via VAS, we found a mean of 92.03% skewing towards highly adherent individuals. This also corresponds to previous results that found a median of 99% with an interquartile range of 10 [46]. Physicians’ estimates resulted in a NA prevalence of 6.4% which is similar to results previously found in other studies, with rates of 3.8% [36] and 9.2% [11]. IS trough level variability displayed a mean of 21.1% in our study which is also comparable to other studies that found CVs% of 18.15% [41], 21.26% [10] and 17.0% [18], respectively. 15.4% of our cohort displayed a CV% > 30. In the study by Rodrigo et al. [20], up to 37.4% of patients were found to have a CV% above 30, though. It must be kept in mind that there is no standardized threshold of CV% that has reliably been associated with adverse outcomes, yet. It still varies considerably in different studies and makes further comparisons challenging [19, 20, 41–43]. Our composite NA score was 40.8%. Other studies that combined different measures reached NA rates up to 70.5% [37], however this specific study displayed high NA rates for each single measure, 46.6% for BAASIS, 38.6% for collateral reports, and 34.1% for sub-therapeutic IS trough levels, respectively. Another study found 26.4% non-adherent and 36.8% partially non-adherent patients when combining self-reports and collateral reports of NA [36]. However, different NA measures were used in both cases.

Our results suggest no agreement between the different NA measures applied at t0 which is well in line with previous studies exhibiting no association between various instruments whatsoever [10, 11, 34, 36]. Especially in case of high trough level variability, no equivalence to other measures is likely. Although NA might contribute to a high variability, many other factors influence this parameter, for instance metabolism, nutrition, drug interactions, and “white-coat adherence” (better adherence before clinic visits) [9, 11]. Our results underscore the assumption that each measurement method functions as a partial indicator for NA and captures different aspects thereof [10, 34].

### Prevalence of electronically monitored and self-reported non-adherence during the study (t1-t6)

During our study, self-reported NA by BAASIS© (two weeks recall) ranged between 9.3% and 20.5% from t1 to t6. When extending the recall period to 4 weeks by adding the respective time points, prevalence rates were 10.8%, 17.1%, and 26.8%, respectively. This rate is lower than at t0 (28.6%) as well as in previously reported literature (27 to 36.6%) [10, 11, 46]. This might be attributable to a potential intervention effect by the study, resulting in a rapid decline of self-reported NA in the first month of the study and a subsequent gradual rise during the following weeks. A comparable effect has been observed in previous studies [28, 29].

Further, self-reported Taking NA remained relatively low from t1 to t6 (1.3–5.6%), indicating that timing deviations of ±2 h constituted the largest part of NA when measured by BAASIS©. When we included timing deviations of ±30 min, NA rose to 38.2–51.4% from t1 to t6. These results are in line with literature displaying much lower rates for Taking NA than Timing NA, as well as only rarely occurring events of drug holiday and dose reductions [36, 46].

Despite these rates of NA, the percentages of self-reported Taking and Timing adherence remained high throughout the whole study. The same occurred for electronically monitored adherence, with a minimum mean of 91.2% for the strictest adherence measure (TM ± 30 min). Previous research [14, 47] found similar high rates of adherence when using EM. NA rates seem to depend on the mode of measure (percentage, count of NA incidents) as well as the definition of the NA threshold (TA, TM ± 2 h, TM ± 30 min respectively) which seem crucial to distinguish when comparing NA rates across studies.

### Associations of self-reported and electronically monitored non-adherence during the study (t1-t6)

The association between self-reported NA and electronically monitored NA in RTRs has only sparsely been examined. Studies undertaking this attempt only received modest results [14, 34]. We used the absolute frequencies of NA incidence for both self-reports and EM in order to receive the highest possible accuracy. Throughout the study, the association between self-reports and EM remained moderate to high. Overall, highest correlations could be found for TM ± 30 min. As suggested by previous literature [32], self-reports and EM seem to produce similar results on NA which leads to the conclusion that they measure the same construct. Since the highest accuracy was reached for the strictest measure of NA (TM ± 30 min), it can be assumed that small deviations from the dosing schedule are generally reported by patients. Lower correlations for TA or TM ± 2 h might occur due to social desirability [8, 13], as major deviations from the medication regimen might rather want to be disguised than smaller ones. However, it was suggested that disclosure of NA to a researcher could be more accurate than to clinical staff [12] and hence our results could also display genuine reports of NA. Low correlations could therefore also result from missed diary entries. In these cases, NA might have been registered by EM, even though the IS was taken from another source [28, 39]. Another possibility is electronic failure, since EM devices may fail to register openings due to technical malfunctions [28] or impaired cellular reception.

We further found self-reported NA (VAS) to be the only significant predictor of the percentage of electronically monitored adherence. This is in line with previous research that also found self-reports to be predictive of electronically monitored NA [12, 14]. IS trough level variability and physicians’ estimates on NA (VAS) seem to play a subordinate role.

### Effect of measurement and change of non-adherence over time (t1-t6)

Our results revealed a steadily decreasing probability of electronically monitored adherence over time, both for TA, TM ± 2 h, and TM ± 30 min. As discussed earlier, this decrease could reflect the waning of an intervention effect induced by the study design. Applying EM as a NA measure was previously associated with an interference of normal drug intake in the first month of monitoring. It was shown that NA was very low in the beginning, but was shortly followed by an increase of NA until it stabilized after 35 to 40 days [28, 29]. In our study the probability of adherence continuously decreased until the end of the study displaying no stabilization of adherence behavior. However, we found a trend towards a significant change in intake behavior after t3 in TM ± 30 min, suggesting a shift in adherence after 4–6 weeks (28–42 days respectively). Our results are not fully comparable to previous studies, though, since we depicted dichotomized adherence behavior during a two week interval, while other research focused on dichotomized intake behavior for each single day [28, 29]. Further, there was no self-assessed intervention effect. This might be due to subtle changes in adherence behavior over a period of three months which were not consciously observed by the respective patients.

### Summary, conclusions and limitations

This study examined the concordance of different adherence measurement methods in RTRs with special focus on prospective measures of self-reported and electronically monitored NA. We found no correlation between IS trough levels, physicians’ estimates, and self-reports. Self-reports however, were found to be moderately to highly concordant with EM which allows for the conclusion that they both measure NA equally. Methodological flaws cannot be excluded for both methods (memory bias, social desirability vs. electronic malfunction, misuse of device [8, 13, 28, 30, 31]), though. We conclude that self-reports are equally accurate as EM, but seem to be superior to EM in clinical practice due to its practicability and economy [8, 14, 15]. EM on the other side can be viewed as a valid tool in research for gaining greater insight in the dynamics of NA [8, 15]. The possibility of an intervention effect induced by EM must be kept in mind when measuring NA. Surveillance of IS intake via EM with intermittent phone calls could improve adherence on a short-term basis. To establish long-term effects, further research is necessary.

Certain limitations of our study need to be mentioned. Although we assessed the consistency of diary use, we did not eliminate cases in which diary keeping was partially incomplete [28]. There might have been incidents in which patients indeed took their medication but forgot to use the diary. For these patients, we rather measured adherence to pillbox and diary use than actual adherence to IS medication. Better diary keeping could be intrinsic to patients that are already more adherent [39] which could increase the gap between adherent and non-adherent patients. In further studies, it is recommendable to evaluate both the patients’ acceptance and intention of future pillbox and diary use before enrollment. If necessary, adequate adjustments to the patient’s routine could be made by replacing pillboxes by pill bottles [39, 48] or diaries by mobile apps. Generalization of results must also be treated with caution. A responder bias towards adherent patients cannot be excluded, since we had a highly adherent population with low variation in data. Replication of our results in a more non-adherent population is necessary. Future studies should also focus more strongly on the influence of biological aspects on trough level variability when using this parameter as a measure of adherence.

## Supplementary information

**Additional file 1.** Percentages of Taking Adherence t1-t6 – Self-Reports

**Additional file 2.** Percentages of Timing Adherence ±2 h t1-t6 – Self-Reports

**Additional file 3.** Percentages of Timing Adherence ±30 min t1-t6 – Self-Reports

**Additional file 4.** Percentages of Taking Adherence t1-t6 – Electronic Monitoring

**Additional file 5.** Percentages of Timing Adherence ±2 h t1-t6 – Electronic Monitoring

**Additional file 6.** Percentages of Timing Adherence ±30 min - Electronic Monitoring

**Additional file 7.** a: Percentage Frequency of Taking Adherence - Electronic Monitoring (raw scores). 8 b: Estimated probability of Taking Adherence - Electronic Monitoring.

**Additional file 8.** a: Percentage Frequency of Timing Adherence ±2 h – Electronic Monitoring (raw scores). 8 b: Estimated probability of Timing Adherence ±2 h – Electronic Monitoring.

**Additional file 9.** a: Course of unadjusted means of the self-assessed intervention effect. 9 b: Boxplots for the self-assessed intervention effect.

## Data Availability

The data supporting our findings can be requested from Dipl.-Psych. Marietta Lieb (marietta.lieb@uk-erlangen.de) and Prof. Yesim Erim (yesim.erim@uk-erlangen.de).

## Abbreviations

TA: Taking Adherence
TM ± 2 h: Timing Adherence ±2 h
TM ± 30 min: Timing Adherence ±30 min
VAS: Visual Analogue Scale
NA: Non-Adherence
IS: Immunosuppressant/immunosuppressive
RTR: Renal Transplant Recipient
LMM: Linear Mixed Model
ICC: Intraclass Correlation Coefficient
ns: Not significant
EM: Electronic Monitoring
