# Supplementary figures and images for: Accuracy and concordance of measurement methods to assess non-adherence after renal transplantation - a prospective study
Source: BMC Nephrol. 2020 Mar 31;21:114. doi: 10.1186/s12882-020-01781-1 (PMC7110822; doi:10.1186/s12882-020-01781-1)

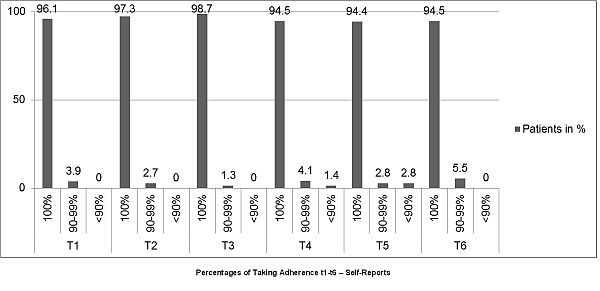

Supplement: Supplementary file 1 — Additional file 1. Percentages of Taking Adherence t1-t6 – Self-Reports [file 12882_2020_1781_MOESM1_ESM.jpg]

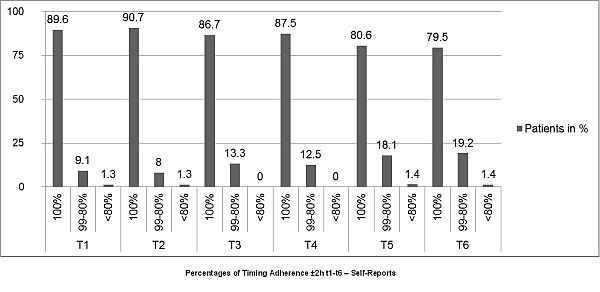

Supplement: Supplementary file 2 — Additional file 2. Percentages of Timing Adherence ±2 h t1-t6 – Self-Reports [file 12882_2020_1781_MOESM2_ESM.jpg]

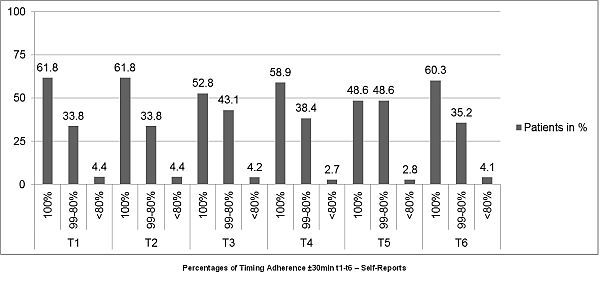

Supplement: Supplementary file 3 — Additional file 3. Percentages of Timing Adherence ±30 min t1-t6 – Self-Reports [file 12882_2020_1781_MOESM3_ESM.jpg]

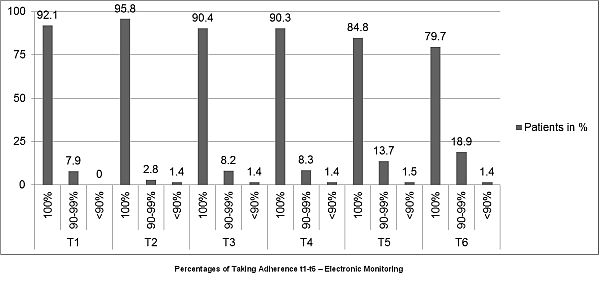

Supplement: Supplementary file 4 — Additional file 4. Percentages of Taking Adherence t1-t6 – Electronic Monitoring [file 12882_2020_1781_MOESM4_ESM.jpg]

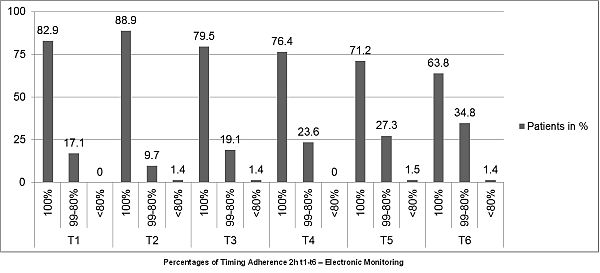

Supplement: Supplementary file 5 — Additional file 5. Percentages of Timing Adherence ±2 h t1-t6 – Electronic Monitoring [file 12882_2020_1781_MOESM5_ESM.jpg]

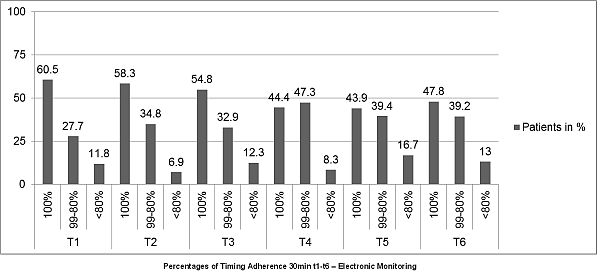

Supplement: Supplementary file 6 — Additional file 6. Percentages of Timing Adherence ±30 min - Electronic Monitoring [file 12882_2020_1781_MOESM6_ESM.jpg]

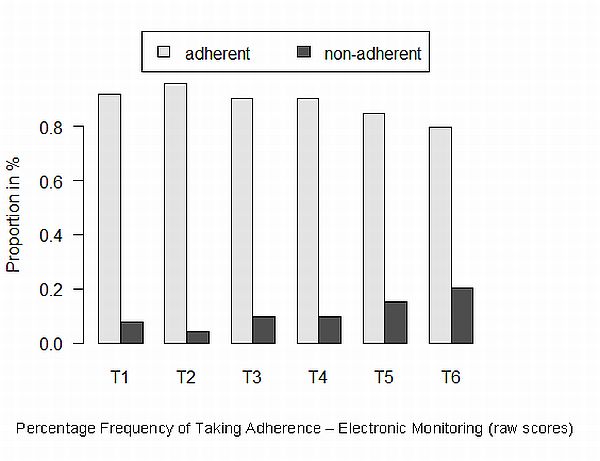

Supplement: Supplementary file 7 — Additional file 7. a: Percentage Frequency of Taking Adherence - Electronic Monitoring (raw scores). 8 b: Estimated probability of Taking Adherence - Electronic Monitoring. [file 12882_2020_1781_MOESM7_ESM.zip › Additional File 7aR3.jpg]

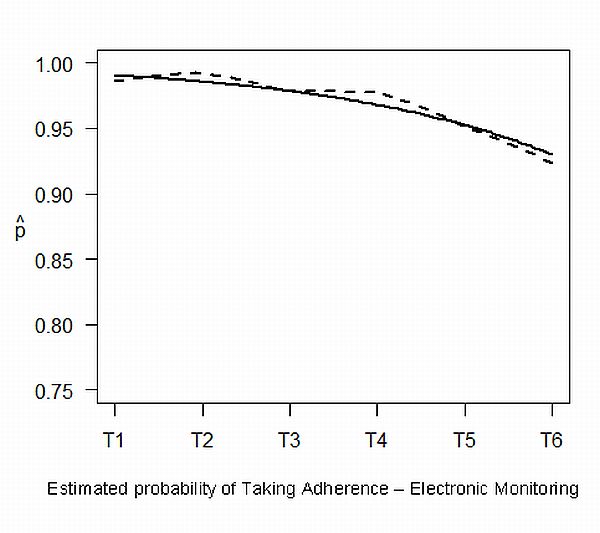

Supplement: Supplementary file 7 — Additional file 7. a: Percentage Frequency of Taking Adherence - Electronic Monitoring (raw scores). 8 b: Estimated probability of Taking Adherence - Electronic Monitoring. [file 12882_2020_1781_MOESM7_ESM.zip › Additional File 7bR3.jpg]

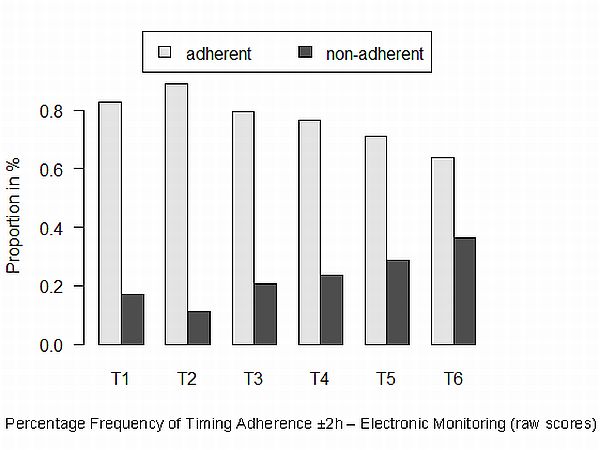

Supplement: Supplementary file 8 — Additional file 8. a: Percentage Frequency of Timing Adherence ±2 h – Electronic Monitoring (raw scores). 8 b: Estimated probability of Timing Adherence ±2 h – Electronic Monitoring. [file 12882_2020_1781_MOESM8_ESM.zip › Additional File 8aR3.jpg]

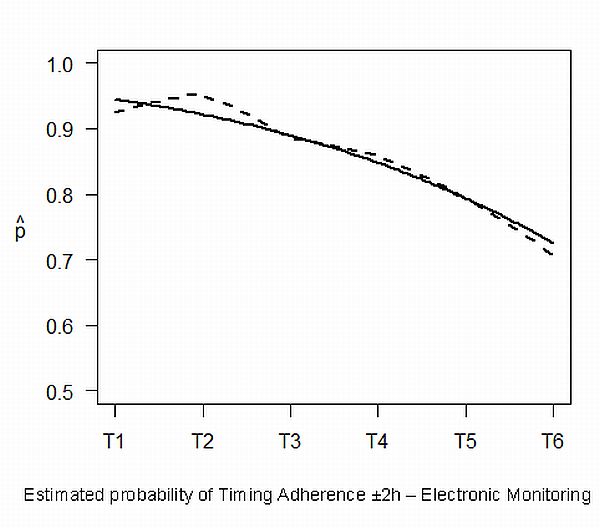

Supplement: Supplementary file 8 — Additional file 8. a: Percentage Frequency of Timing Adherence ±2 h – Electronic Monitoring (raw scores). 8 b: Estimated probability of Timing Adherence ±2 h – Electronic Monitoring. [file 12882_2020_1781_MOESM8_ESM.zip › Additional File 8bR3.jpg]

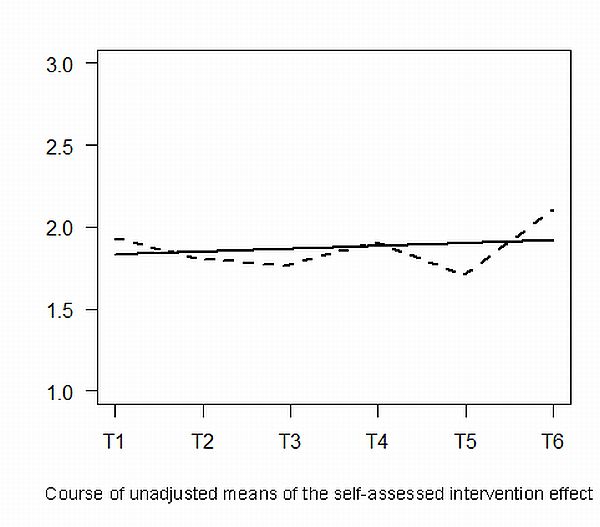

Supplement: Supplementary file 9 — Additional file 9. a: Course of unadjusted means of the self-assessed intervention effect. 9 b: Boxplots for the self-assessed intervention effect. [file 12882_2020_1781_MOESM9_ESM.zip › Additional File 9aR3.jpg]

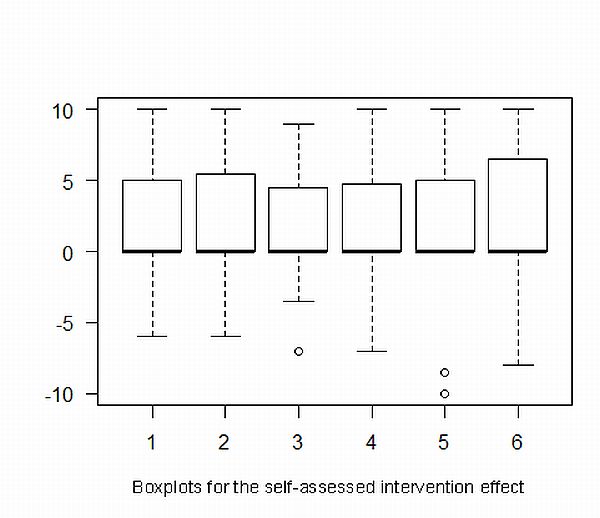

Supplement: Supplementary file 9 — Additional file 9. a: Course of unadjusted means of the self-assessed intervention effect. 9 b: Boxplots for the self-assessed intervention effect. [file 12882_2020_1781_MOESM9_ESM.zip › Additional File 9bR3.jpg]
